# Association of Serum 1,25-Dihydroxyvitamin D Levels with Erythropoietin Resistance Index-Based Relative Erythropoiesis-Stimulating Agent Hyporesponsiveness in Pre-Dialysis Chronic Kidney Disease

**DOI:** 10.3390/ijms27188044

**Published:** 2026-09-10

**Authors:** Jun Yeong Kim, Dong Won Lee, Soo Bong Lee, Il Young Kim

**Affiliations:** 1 Department of Internal Medicine, Pusan National University School of Medicine, Yangsan 50612, Republic of Korea; jyk42313@naver.com (J.Y.K.); dongwonlee@pusan.ac.kr (D.W.L.); sbleemd@pusan.ac.kr (S.B.L.); 2 Research Institute for Convergence of Biomedical Science and Technology, Pusan National University Yangsan Hospital, Yangsan 50612, Republic of Korea

**Keywords:** anemia, chronic kidney disease, erythropoietin resistance index, vitamin D

## Abstract

Erythropoiesis-stimulating agent (ESA) hyporesponsiveness is a major challenge in the management of anemia in chronic kidney disease (CKD). This study evaluated the association between serum 1,25-dihydroxyvitamin D [1,25(OH)_2_D] levels and ESA responsiveness in patients with pre-dialysis CKD. This retrospective cohort study included 363 adult patients with anemia and pre-dialysis CKD who received continuous erythropoietin receptor activator therapy for at least three months. ESA responsiveness was assessed using the erythropoietin resistance index (ERI). The highest ERI tertile was used to define ERI-based relative ESA hyporesponsiveness. Among 363 patients, 121 (33.3%) were classified as having relative ESA hyporesponsiveness. Serum 1,25(OH)_2_D levels were significantly lower in the relative ESA-hyporesponsive group. In multivariable analyses, lower serum 1,25(OH)_2_D levels were independently associated with relative ESA hyporesponsiveness and higher log-transformed ERI. Exploratory ROC analysis showed modest discriminative ability for identifying patients in the highest ERI tertile. Lower serum 1,25(OH)_2_D levels were independently associated with ERI-based relative ESA hyporesponsiveness in pre-dialysis CKD. These findings suggest that active vitamin D status may be linked to ESA responsiveness, as assessed by ERI.

## 1. Introduction

Anemia is one of the most frequent complications of CKD [1]. In this population, impaired renal function and anemia are closely associated with increased mortality and a higher prevalence of CVD [2]. Although renal anemia may be influenced by chronic inflammation, iron deficiency, and other factors, insufficient erythropoietin production is considered the dominant pathogenic mechanism. Therefore, treatment strategies for CKD-related anemia generally include ESAs in combination with iron supplementation [3]. However, a substantial proportion of patients, including nearly 15% of those undergoing dialysis, fail to achieve target hemoglobin concentrations despite receiving high-dose ESA therapy [4].

The 2026 KDIGO guidelines describe ESA hyporesponsiveness as either failure to reach the target hemoglobin level despite an adequate ESA dose or the need for high ESA doses to maintain the desired hemoglobin concentration [5]. ESA hyporesponsiveness and exposure to higher ESA doses have been linked to poor outcomes in CKD, including infection, hospitalization, and cardiovascular mortality [6]. Thus, identifying potentially modifiable contributors to inadequate ESA response is essential for improving the effectiveness and safety of anemia management in patients with CKD [7].

Abnormal vitamin D metabolism is highly prevalent in CKD because of reduced 25(OH)D availability and diminished 1α-hydroxylase activity, which limits conversion of 25(OH)D to 1,25(OH)_2_D [8]. Beyond its classical role in mineral metabolism and bone health, vitamin D has pleiotropic effects in various tissues and has been associated with several chronic disorders, including diabetes, hypertension, CVD, and multiple types of cancer [9]. Although previous studies have suggested a link between vitamin D status and anemia, its association with ESA responsiveness in CKD remains insufficiently understood [10]. Accordingly, this study aimed to evaluate the association between circulating 1,25(OH)_2_D levels and ERI-based relative ESA hyporesponsiveness in patients with pre-dialysis CKD.

## 2. Results

### 2.1. Clinical and Laboratory Characteristics of the Study Subjects

The clinical and laboratory characteristics of the study cohort are summarized in Table 1. A total of 363 patients were included in the final analysis. Of these, 121 patients (33.3%) were classified into the highest ERI tertile and were defined as having relative ESA hyporesponsiveness, whereas 242 (66.7%) were classified as non-hyporesponsive. The median age was 51 years (IQR, 37–67), 52.6% were male, and 47.9% had diabetes mellitus.

The relative ESA-hyporesponsive group showed significantly lower serum 1,25(OH)_2_D levels than the non-hyporesponsive group [11.9 pg/mL (IQR, 8.1–15.2) vs. 17.8 pg/mL (IQR, 12.5–27.0), *p* < 0.001]. Hemoglobin, transferrin saturation, ferritin, 1,25(OH)_2_D levels, and eGFR were significantly lower in the relative ESA-hyporesponsive group, whereas serum iPTH, continuous erythropoietin receptor activator (CERA; methoxy polyethylene glycol-epoetin beta) dose, ERI, and hsCRP were significantly higher in this group. Age, sex, diabetes status, BMI, blood pressure, albumin, calcium, phosphate, total cholesterol, 25(OH)D, and urinary albumin did not differ significantly between the two groups.

### 2.2. Association Between Serum 1,25(OH)_2_D Level and ERI-Based Relative ESA Hyporesponsiveness

Table 2 presents the associations of clinical and laboratory factors with relative ESA hyporesponsiveness. In univariable logistic regression analysis, serum 1,25(OH)_2_D was significantly associated with relative ESA hyporesponsiveness (per 1 pg/mL; odds ratio [OR], 0.88; 95% CI, 0.85–0.92; *p* < 0.001), whereas serum 25(OH)D was not. Relative ESA hyporesponsiveness was also associated with transferrin saturation (per 1%; OR, 0.92; 95% CI, 0.90–0.95; *p* < 0.001), ferritin (per 10 ng/mL; OR, 0.96; 95% CI, 0.94–0.98; *p* < 0.001), serum iPTH (per 10 pg/mL; OR, 1.14; 95% CI, 1.09–1.19; *p* < 0.001), eGFR (per 1 mL/min/1.73 m^2^; OR, 0.96; 95% CI, 0.94–0.98; *p* < 0.001), and hsCRP (per 1 mg/dL; OR, 3.11; 95% CI, 1.91–5.06; *p* < 0.001).

In the multivariable model, serum 1,25(OH)_2_D remained independently associated with relative ESA hyporesponsiveness (per 1 pg/mL; OR, 0.88; 95% CI, 0.85–0.92; *p* < 0.001). Transferrin saturation (per 1%; OR, 0.93; 95% CI, 0.90–0.97; *p* < 0.001), ferritin (per 10 ng/mL; OR, 0.97; 95% CI, 0.95–0.99; *p* = 0.001), serum iPTH (per 10 pg/mL; OR, 1.08; 95% CI, 1.02–1.13; *p* = 0.007), and hsCRP (per 1 mg/dL; OR, 2.35; 95% CI, 1.38–4.01; *p* = 0.002) also showed independent associations with relative ESA hyporesponsiveness. Variance inflation factors ranged from 1.03 to 1.25, indicating no evidence of significant multicollinearity among the included covariates.

**Figure 1:**
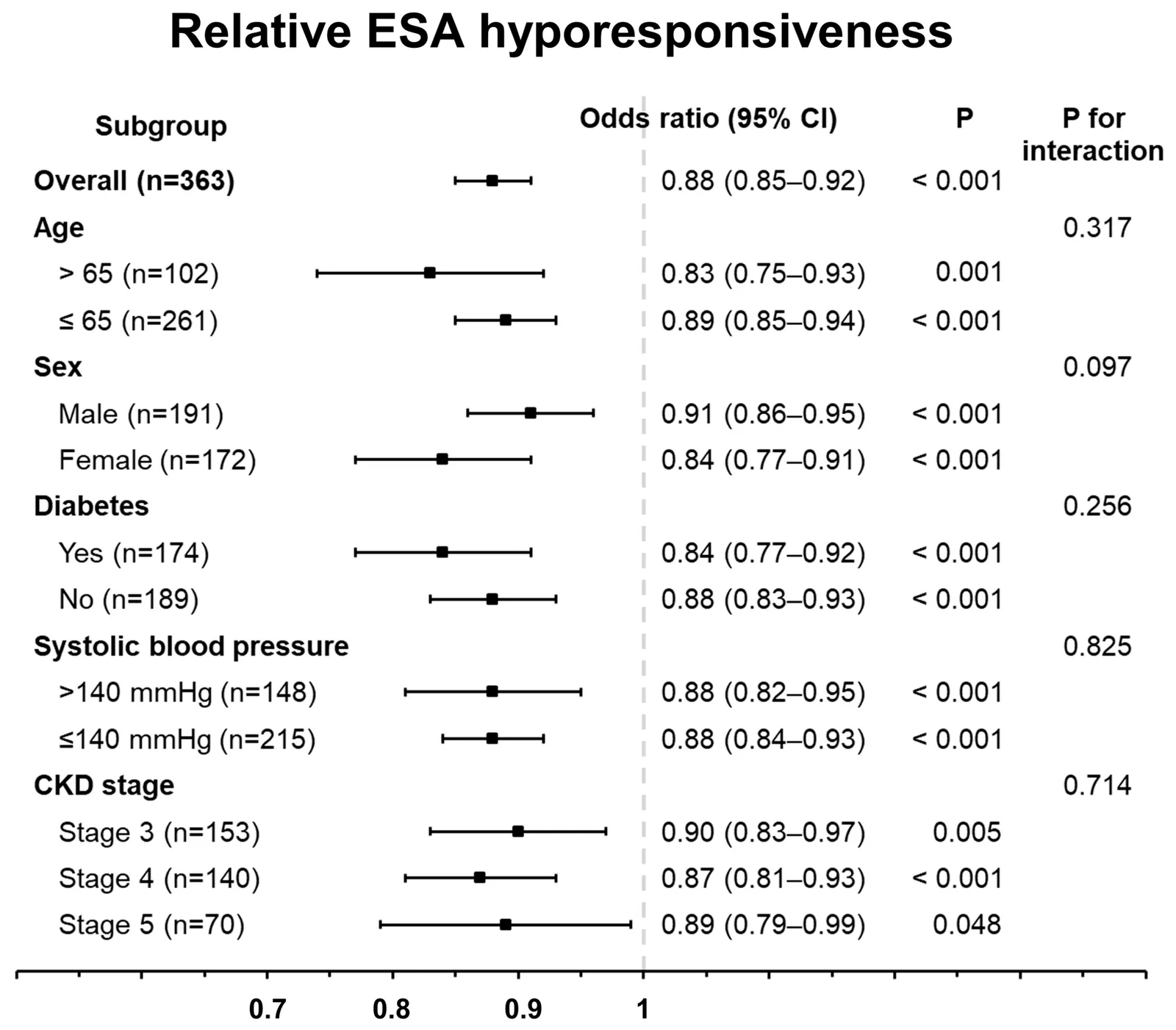
Association between serum 1,25(OH)_2_D levels and ERI-based relative ESA hyporesponsiveness across predefined subgroups. Odds ratios for relative ESA hyporesponsiveness associated with serum 1,25(OH)_2_D levels are presented according to age (>65 or ≤65 years), sex, systolic blood pressure (>140 or ≤140 mmHg), diabetes mellitus status, and CKD stage (stages 3, 4, and 5). Multivariable logistic regression analysis showed that the inverse association between serum 1,25(OH)_2_D levels and relative ESA hyporesponsiveness was broadly similar across these subgroups. *p* values for interaction were calculated by including interaction terms between serum 1,25(OH)_2_D levels and each subgroup variable.

Subgroup analyses were performed to assess the relationship between serum 1,25(OH)_2_D and relative ESA hyporesponsiveness according to age, sex, systolic blood pressure, CKD stage, and diabetes status (Figure 1). The inverse association between serum 1,25(OH)_2_D levels and relative ESA hyporesponsiveness was broadly similar across predefined subgroups, including patients aged > 65 and ≤65 years, men and women, those with systolic blood pressure > 140 and ≤140 mmHg, patients with and without diabetes mellitus, and patients with CKD stages 3, 4, and 5. No statistically significant interactions were observed between serum 1,25(OH)_2_D levels and these subgroup variables.

### 2.3. Association Between Serum 1,25(OH)_2_D Level and ERI

Table 3 presents the relationships between clinical and laboratory parameters and ERI. In univariable correlation analyses using Spearman’s rank correlation coefficients, ERI showed significant negative correlations with transferrin saturation (ρ = −0.25, *p* < 0.001), ferritin (ρ = −0.18, *p* < 0.001), eGFR (ρ = −0.28, *p* < 0.001), and serum 1,25(OH)_2_D levels (ρ = −0.45, *p* < 0.001). In contrast, ERI was positively correlated with serum iPTH (ρ = 0.34, *p* < 0.001) and hsCRP levels (ρ = 0.36, *p* < 0.001). No significant correlation was observed between ERI and serum 25(OH)D levels (ρ = 0.04, *p* = 0.477).

In multivariable linear regression analysis using log-transformed ERI as the dependent variable, serum 1,25(OH)_2_D remained independently associated with ERI (β = −0.36, *p* < 0.001). Transferrin saturation (β = −0.18, *p* < 0.001), ferritin (β = −0.13, *p* = 0.005), serum iPTH (β = 0.18, *p* < 0.001), and hsCRP (β = 0.15, *p* = 0.001) were also independently associated with log-transformed ERI.

### 2.4. Exploratory ROC Analysis of Serum 1,25(OH)_2_D for ERI-Based Relative ESA Hyporesponsiveness

**Figure 2:**
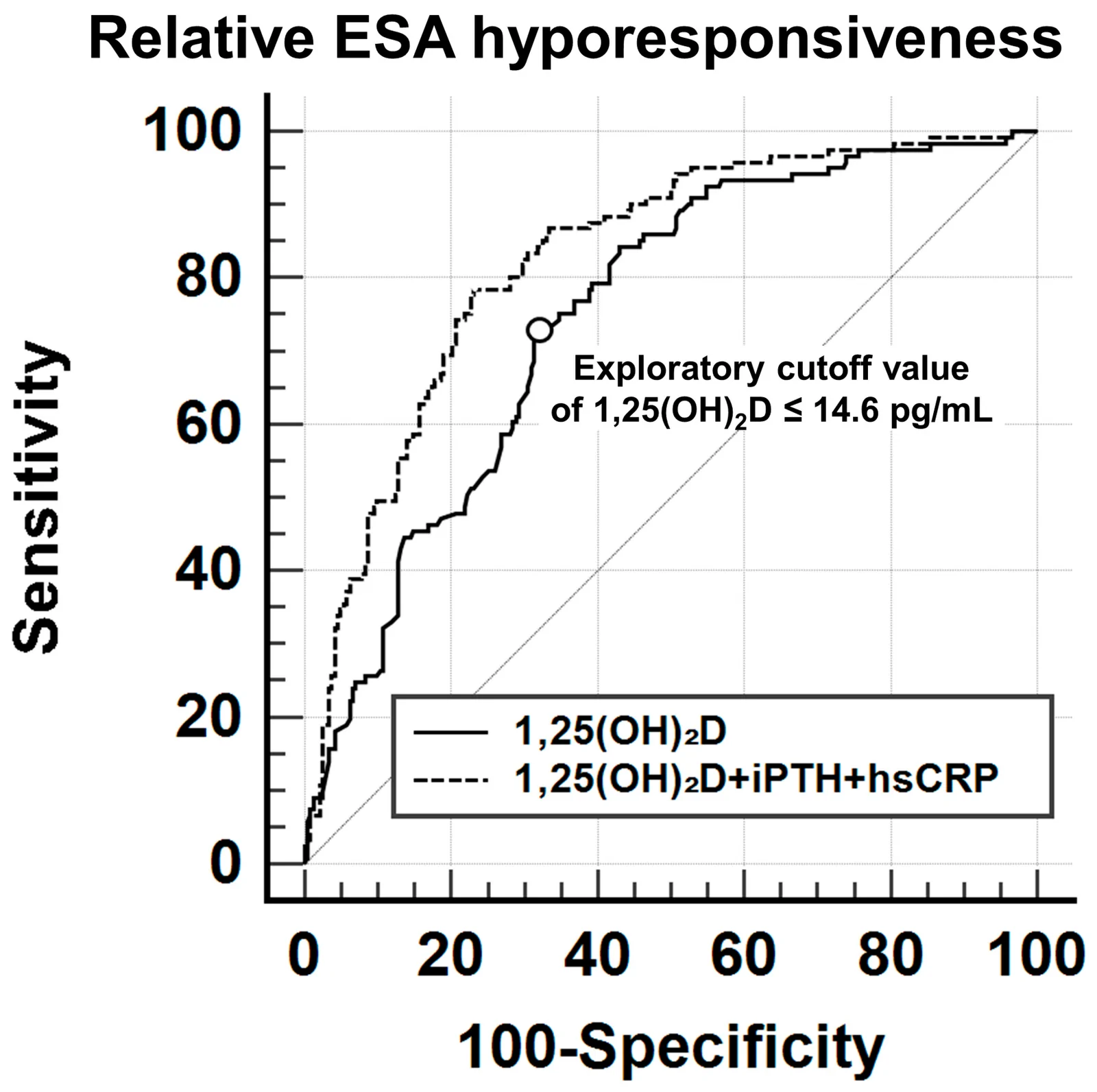
Exploratory ROC analysis of serum 1,25(OH)_2_D for discriminating ERI-based relative ESA hyporesponsiveness within the study cohort. ROC curves were used to explore the ability of serum 1,25(OH)_2_D to identify patients in the highest ERI tertile among the study population (*n* = 363). The AUC for serum 1,25(OH)_2_D was 0.749 (95% CI, 0.701–0.793). The exploratory cutoff value determined by the Youden index in this cohort was ≤14.6 pg/mL, with a sensitivity of 73.6% and a specificity of 68.2%. This cutoff should be interpreted as exploratory and should not be used as a clinically validated diagnostic threshold for ESA hyporesponsiveness. An exploratory combined model incorporating serum 1,25(OH)_2_D, iPTH, and hsCRP showed significantly better discriminative performance than serum 1,25(OH)_2_D alone [AUC, 0.827 (95% CI, 0.784–0.864) vs. 0.749 (95% CI, 0.701–0.793); *p* < 0.001].

Exploratory ROC curve analysis was performed to evaluate the ability of serum 1,25(OH)*_2_*D to discriminate relative ESA hyporesponsiveness within this cohort (Figure 2). Serum 1,25(OH)*_2_*D showed an AUC of 0.749 (95% CI, 0.701–0.793), indicating modest discriminative ability for identifying patients in the highest ERI tertile. The exploratory cutoff value determined by the Youden index in this cohort was ≤14.6 pg/mL, with a sensitivity of 73.6% (95% CI, 64.8–81.2%) and a specificity of 68.2% (95% CI, 62.0–74.0%). This cutoff should be interpreted as exploratory and should not be regarded as a clinically validated diagnostic threshold for ESA hyporesponsiveness. An exploratory combined model incorporating serum 1,25(OH)*_2_*D, iPTH, and hsCRP showed a higher AUC than serum 1,25(OH)*_2_*D alone [0.827 (95% CI, 0.784–0.864) vs. 0.749 (95% CI, 0.701–0.793), *p* < 0.001].

## 3. Discussion

The present study demonstrated that lower serum 1,25(OH)_2_D levels were independently associated with ERI-based relative ESA hyporesponsiveness in patients with anemia and pre-dialysis CKD treated with CERA. This association remained significant after adjustment for iron status, inflammation, secondary hyperparathyroidism, kidney function, and other clinically relevant factors. Serum 1,25(OH)_2_D levels were also independently and inversely associated with log-transformed ERI, supporting a consistent relationship between lower active vitamin D status and greater ESA resistance. These findings suggest that active vitamin D status may be linked to ESA responsiveness, as reflected by ERI, in patients with pre-dialysis CKD. Exploratory ROC analysis suggested modest discriminative ability of serum 1,25(OH)_2_D, but its clinical utility requires further validation.

ESA hyporesponsiveness in CKD is influenced by a broad range of clinical and biological factors. In addition to insufficient endogenous erythropoietin production, absolute or functional iron deficiency is recognized as a leading cause of inadequate response to ESA therapy [11]. Chronic inflammation can further reduce ESA responsiveness by increasing proinflammatory cytokines, such as interleukin-1 and interleukin-6, which inhibit erythroid progenitor cell proliferation and differentiation [12]. Secondary hyperparathyroidism may also compromise erythropoiesis by inducing bone marrow fibrosis and disrupting the hematopoietic microenvironment [12]. Other potential contributors include vitamin B12 or folate deficiency, retention of uremic toxins, and treatment with certain medications, such as angiotensin-converting enzyme inhibitors [13,14]. Despite accumulating evidence linking vitamin D status to anemia, the association between serum 1,25(OH)_2_D levels and ESA hyporesponsiveness among patients with CKD remains uncertain. In this context, the present findings provide additional evidence that active vitamin D status may be related to ESA responsiveness assessed by ERI in patients with pre-dialysis CKD.

The relationship between vitamin D deficiency and anemia has been examined in several CKD cohorts, but evidence regarding vitamin D status and ESA responsiveness remains limited. Kendrick et al., in a large analysis of 16,301 patients with pre-dialysis CKD, showed that lower 25(OH)D levels were independently linked to lower hemoglobin concentrations [15]. In another study of 410 patients with end-stage renal disease, Kim et al. reported that reduced 25(OH)D levels were associated with a higher risk of anemia [16]. Patel et al. further demonstrated, in 1661 CKD patients, that lower concentrations of both 25(OH)D and 1,25(OH)_2_D were related to lower hemoglobin levels [17]. Compared with the evidence on anemia, data regarding vitamin D status and ESA hyporesponsiveness are scarce. Kiss et al. previously reported that lower 25(OH)D levels were associated with greater ESA resistance in 142 hemodialysis patients [18]. However, the generalizability of that study may be limited by its modest sample size and its restriction to dialysis patients. The present study extends previous findings by evaluating a relatively larger cohort of patients with pre-dialysis CKD treated with CERA and by focusing on serum 1,25(OH)_2_D, the biologically active form of vitamin D.

Several mechanisms may explain the observed association between low serum 1,25(OH)_2_D levels and relative ESA hyporesponsiveness. First, inflammation may be involved, as chronic inflammation is a major contributor to impaired ESA response. Vitamin D has important regulatory effects on immune function. In CKD, reduced 1,25(OH)_2_D levels may be associated with immune activation and increased release of proinflammatory cytokines, which can inhibit erythroid progenitor cell proliferation and differentiation [19]. Immune activation can also increase hepcidin production, which reduces iron availability by sequestering iron within macrophages and leads to functional iron deficiency [20]. These processes may be involved in both anemia and reduced ESA responsiveness in CKD. In the present study, elevated hsCRP was independently associated with relative ESA hyporesponsiveness, supporting the role of inflammation in inadequate ESA response. Second, 1,25(OH)_2_D may affect ESA responsiveness by modulating secondary hyperparathyroidism. Hyperparathyroidism is an established contributor to CKD-related anemia [21]. In CKD, declining 1,25(OH)_2_D levels stimulate a compensatory increase in PTH. Persistent PTH elevation induces bone remodeling, impaired mineralization, and bone loss, which may disrupt the bone marrow microenvironment and suppress the proliferation and maturation of erythroid progenitor cells [22]. Because 1,25(OH)_2_D can suppress PTH synthesis through VDR-mediated regulation of PTH gene transcription in the parathyroid glands, active vitamin D status may be linked to anemia partly through secondary hyperparathyroidism. The independent association between higher iPTH levels and relative ESA hyporesponsiveness observed in this study further supports this potential pathway. Third, experimental evidence suggests that vitamin D may influence erythropoiesis through VDR-mediated actions on erythroid cells, independent of its effects on inflammation and parathyroid hormone regulation [23]. VDR is expressed in hematopoietic stem cells and early erythroid progenitors. After binding to VDR, 1,25(OH)_2_D may act as a transcriptional regulator and induce genes involved in erythroid lineage commitment, proliferation, and maturation. This genomic effect may act synergistically with erythropoietin and thereby facilitate erythropoiesis [23]. These observations suggest a potential biological link between active vitamin D status and erythropoiesis beyond its anti-inflammatory and PTH-suppressive effects. Importantly, in the multivariable model, serum 1,25(OH)_2_D remained independently associated with relative ESA hyporesponsiveness even after adjustment for iPTH and hsCRP, further supporting an association between active vitamin D status and ESA responsiveness.

Although multiple determinants of inadequate ESA response have been identified, reduced ESA responsiveness often remains unexplained in clinical practice. The present findings suggest that serum 1,25(OH)_2_D levels may help characterize ESA responsiveness assessed by ERI in patients with CKD, although their clinical utility requires further validation. Vitamin D therapy is commonly used to treat CKD-MBD [24], but its effect on CKD-related anemia remains uncertain. Previous studies evaluating the hematologic impact of vitamin D supplementation have yielded conflicting results. A recent meta-analysis by Ahmad et al. showed that vitamin D supplementation was associated with a significant increase in hemoglobin levels in hemodialysis patients with CKD, supporting its potential benefit in CKD-associated anemia [25]. In contrast, a systematic review and meta-analysis of randomized controlled trials by Arabi et al. reported no significant changes in hemoglobin or ferritin levels, although modest improvements in transferrin saturation and iron status were observed [26]. These divergent findings suggest that the effect of vitamin D therapy on anemia may vary according to initial vitamin D status, CKD stage, dialysis status, patient characteristics, type and duration of vitamin D treatment, and concomitant anemia therapy. Further prospective studies are warranted to determine whether interventions targeting active vitamin D status can improve anemia or ESA responsiveness in patients with pre-dialysis CKD.

Several limitations should be acknowledged. First, the retrospective nature of this study limits the ability to infer causality between low serum 1,25(OH)_2_D levels and ERI-based relative ESA hyporesponsiveness. Although serum 25(OH)D and 1,25(OH)_2_D levels were assessed at the time of CERA initiation, subsequent changes in vitamin D status, mineral metabolism, inflammation, iron parameters, or clinical condition during treatment could not be fully captured. Therefore, the observed association should not be interpreted as evidence that low active vitamin D directly causes ESA hyporesponsiveness. Second, this was a single-center study, which may limit the generalizability of the findings. In addition, patients receiving vitamin D supplements, phosphate binders, or calcimimetics were excluded to minimize the potential confounding effects of CKD-MBD treatment on endogenous vitamin D status. However, these exclusions may have introduced selection bias, particularly among patients with advanced CKD, in whom such medications are more commonly prescribed. Therefore, the study population may not fully represent the broader population of patients with advanced pre-dialysis CKD. Third, residual confounding from unmeasured factors remains possible. For example, alternative causes of anemia, such as vitamin B12 or folate deficiency, occult infection, nutritional status, and treatment-related changes, were not systematically evaluated. In addition, fibroblast growth factor 23, which may suppress 1α-hydroxylase activity and thereby influence circulating 1,25(OH)_2_D levels, was not available in this retrospective dataset. Therefore, we could not determine whether the association between serum 1,25(OH)_2_D levels and relative ESA hyporesponsiveness persisted after adjustment for fibroblast growth factor 23. Fourth, serum 25(OH)D and 1,25(OH)_2_D levels were measured only once at the time of CERA initiation. Because vitamin D concentrations can vary over time depending on season, nutritional status, kidney function, and clinical condition, a single measurement may not fully represent long-term vitamin D status. Fifth, ERI-based relative ESA hyporesponsiveness was defined using the highest ERI tertile rather than an established absolute clinical threshold based on failure to achieve or maintain target hemoglobin levels despite high-dose ESA therapy. Therefore, the findings should be interpreted as reflecting relative ESA hyporesponsiveness within this cohort rather than established clinical ESA hyporesponsiveness.

Despite these limitations, this study has several strengths. First, serum 1,25(OH)_2_D, rather than 25(OH)D, was measured. This distinction is clinically meaningful because 1,25(OH)_2_D is the biologically active form of vitamin D that directly binds to VDR and mediates hormonal effects, whereas 25(OH)D primarily reflects vitamin D storage status. Thus, the association between serum 1,25(OH)_2_D and relative ESA hyporesponsiveness observed in this study may offer a more physiologically relevant assessment than previous studies relying only on 25(OH)D. Second, the study was conducted in patients with pre-dialysis CKD. Earlier studies mostly focused on 25(OH)D or included dialysis-dependent patients, whereas the present study specifically evaluated the relationship between serum 1,25(OH)_2_D and relative ESA hyporesponsiveness in a pre-dialysis CKD population. Third, several potential confounders were minimized by enrolling ESA-naïve patients without overt iron deficiency anemia and by excluding patients who had received red blood cell transfusion, those with malignancy or active infection, and those receiving treatments that could alter vitamin D metabolism.

## 4. Materials and Methods

### 4.1. Study Population

This retrospective study included 363 patients who visited Pusan National University Yangsan Hospital from January 2010 to December 2019. Adult patients aged ≥ 19 years were eligible if they had anemia and CKD, defined as an eGFR of <60 mL/min/1.73 m^2^. Patients undergoing renal replacement therapy were excluded. The eGFR was estimated using the CKD-EPI equation. Anemia was defined as a hemoglobin level of <12 g/dL in women and <13 g/dL in men [5]. Patients were included if they were ESA-naïve at the time of CERA initiation and had received CERA (Mircera, Roche, Basel, Switzerland) therapy for at least three months. Patients treated with ESAs other than CERA were excluded. To reduce the confounding effect of iron deficiency on ESA responsiveness, patients with transferrin saturation < 20% or ferritin < 100 ng/mL were excluded. Patients who had received red blood cell transfusion during the 3-month CERA treatment period were also excluded. In addition, patients with malignancy at the time of CERA initiation or active infection during the 3-month CERA treatment period were excluded. Patients receiving vitamin D supplements, calcimimetics, or phosphate binders were also excluded because these treatments may alter endogenous vitamin D levels.

### 4.2. Study Variables

Demographic and clinical characteristics, such as age, sex, and diabetes status, were retrieved from the patients’ medical records. BMI was calculated as weight divided by height squared and expressed in kg/m^2^. Blood pressure was measured in the right arm using an automated sphygmomanometer after a 5-min rest, with each patient seated comfortably. Diabetes mellitus was defined as the use of antidiabetic medication, fasting plasma glucose ≥ 126 mg/dL, or hemoglobin A1c ≥ 6.5%. Clinical and laboratory data were collected from medical records. Laboratory parameters, including serum albumin, calcium, phosphate, total cholesterol, hemoglobin, hsCRP, iPTH, ferritin, transferrin saturation, serum 25(OH)D, and serum 1,25(OH)_2_D, were assessed at the time of CERA initiation. The same 3-month period after CERA initiation was used to calculate the mean CERA dose, mean hemoglobin concentration, and ERI. Urinary albumin was assessed using the urinary albumin-to-creatinine ratio (mg/g creatinine). Serum 25(OH)D levels were analyzed by chemiluminescent immunoassay (DiaSorin, Saluggia, Italy; reference range, 4.8–52.8 ng/mL), and serum 1,25(OH)_2_D levels were measured using radioimmunoassay (DIAsource ImmunoAssays, Louvain-la-Neuve, Belgium; reference range, 19.6–54.3 pg/mL).

### 4.3. Definition of ESA Hyporesponsiveness

ESA hyporesponsiveness was assessed using the ERI. The ERI was calculated by first dividing the mean monthly CERA dose (µg/month) during the first three months after treatment initiation by body weight at CERA initiation to obtain the body-weight-adjusted CERA dose (µg/kg/month), and then dividing this value by the mean hemoglobin concentration (g/dL) during the same 3-month period. During the 3-month treatment period, CERA dose and dosing interval were adjusted according to hemoglobin concentration, the rate of hemoglobin change, and the treating physician’s clinical judgment. The mean monthly CERA dose during this period was used to calculate the ERI to reflect cumulative ESA exposure. Because no universally accepted absolute ERI threshold is available for defining ESA hyporesponsiveness in CERA-treated patients with pre-dialysis CKD, the highest tertile of ERI was used to operationally define ERI-based relative ESA hyporesponsiveness within the study cohort. This definition was intended to identify patients with relatively poorer ESA responsiveness within the cohort and was not intended to represent a validated clinical threshold for ESA hyporesponsiveness. Patients in the highest ERI tertile were classified as the relative ESA-hyporesponsive group, whereas those in the lower two tertiles were classified as the non-hyporesponsive group.

### 4.4. Statistical Analysis

Continuous variables are expressed as medians and IQRs, and categorical variables are presented as numbers and percentages. Differences between groups were assessed using the Mann–Whitney U test for continuous variables and the chi-square test for categorical variables, as appropriate. Univariable and multivariable logistic regression analyses were performed to identify factors associated with ERI-based relative ESA hyporesponsiveness. Logistic regression results are reported as ORs and 95% CIs. Variables with a *p* value < 0.10 in the univariable analyses were considered for inclusion in the multivariable models. Age and sex were included a priori as clinically relevant demographic covariates, regardless of their univariable associations. The final multivariable logistic regression model included age, sex, transferrin saturation, ferritin, iPTH, serum 1,25(OH)_2_D, eGFR, and hsCRP. Hemoglobin concentration and CERA dose were not included as covariates in the multivariable models because they are components of the ERI calculation. Multicollinearity among covariates was assessed using variance inflation factors. Subgroup analyses were performed to evaluate the association between serum 1,25(OH)_2_D levels and ERI-based relative ESA hyporesponsiveness according to age, sex, systolic blood pressure, diabetes mellitus status, and CKD stage. *p* values for interaction were calculated by including interaction terms between serum 1,25(OH)_2_D levels and each subgroup variable in the multivariable logistic regression models.

Because ERI showed a right-skewed distribution, correlations between ERI and clinical or laboratory variables were assessed using Spearman’s rank correlation coefficients (ρ). Multivariable linear regression analysis was performed using natural-log-transformed ERI as the dependent variable to account for the skewed distribution of ERI. The multivariable linear regression model included age, sex, transferrin saturation, ferritin, iPTH, serum 1,25(OH)_2_D, eGFR, and hsCRP as covariates. Standardized regression coefficients (β) are reported for the linear regression analyses.

Exploratory ROC curve analysis was performed to estimate the AUC for serum 1,25(OH)_2_D in discriminating ERI-based relative ESA hyporesponsiveness. The Youden index was used to determine an exploratory cutoff value in this cohort. For combined models, logistic regression was used to generate predicted probabilities, which were then used to construct ROC curves. AUCs were compared using the method of DeLong et al. [27]. The combined exploratory ROC model was constructed using serum 1,25(OH)_2_D, iPTH, and hsCRP because these variables were biologically relevant to vitamin D metabolism, secondary hyperparathyroidism, and inflammation, respectively, and showed independent associations with relative ESA hyporesponsiveness in the multivariable logistic regression analysis. This model was exploratory and data-informed rather than prespecified.

Statistical significance was set at *p* < 0.05. All analyses were performed using SPSS version 28.0 (SPSS, Inc., Chicago, IL, USA) and MedCalc Statistical Software version 22.023 (MedCalc Software, Ostend, Belgium).

## 5. Conclusions

In conclusion, lower serum 1,25(OH)_2_D levels were independently associated with ERI-based relative ESA hyporesponsiveness in patients with pre-dialysis CKD. This association remained significant after adjustment for iron status, inflammation, secondary hyperparathyroidism, and other clinically relevant factors. Serum 1,25(OH)_2_D was also inversely associated with the erythropoietin resistance index, suggesting a potential role of active vitamin D status in ESA responsiveness. Further prospective and mechanistic studies are needed to determine whether interventions targeting active vitamin D status can improve anemia management in this population.

## Figures and Tables

**Table 1:** Clinical and laboratory characteristics of the study subjects (*n* = 363).

Variables	Total	Relative ESA-Hyporesponsive Group	Non-Hyporesponsive Group	*p*
No. of subjects	363	121 (33.3%)	242 (66.7%)	
Age (years)	51 (37–67)	51 (38–65)	51 (36–68)	0.693
Sex (male)	191 (52.6%)	66 (54.5%)	125 (51.7%)	0.683
Diabetes mellitus	174 (47.9%)	63 (52.1%)	111 (45.9%)	0.316
BMI (kg/m^2^)	24.8 (19.2–30.4)	25.5 (19.2–31.0)	24.1 (19.4–29.9)	0.489
SBP (mmHg)	130 (102–156)	129 (102–152)	131 (103–159)	0.617
DBP (mmHg)	74 (61–92)	76 (62–91)	73 (59–93)	0.447
Serum albumin (g/dL)	4.0 (3.7–4.2)	4.0 (3.7–4.3)	4.0 (3.7–4.1)	0.186
Serum calcium (mg/dL)	8.9 (8.7–9.2)	9.0 (8.7–9.2)	8.9 (8.7–9.2)	0.452
Serum phosphate (mg/dL)	3.5 (3.1–4.2)	3.6 (3.0–4.2)	3.5 (3.1–4.2)	0.980
Total cholesterol (mg/dL)	236 (182–292)	250 (203–297)	230 (178–291)	0.177
Hemoglobin (g/dL)	9.8 (8.1–11.4)	9.0 (7.4–10.1)	10.3 (8.6–11.7)	<0.001
Transferrin saturation (%)	31.7 (26.1–39.5)	28.7 (27.0–31.9)	35.5 (25.7–44.5)	<0.001
Ferritin (ng/mL)	276 (179–412)	220 (187–303)	330 (177–452)	<0.001
Serum iPTH (pg/mL)	81 (64–140)	141 (73–174)	75 (60–120)	<0.001
CERA dose (µg/month)	100 (75–150)	150 (120–200)	75 (60–120)	<0.001
Serum 25(OH)D (ng/mL)	12.8 (9.7–18.8)	13.6 (10.2–18.6)	12.7 (9.3–18.8)	0.190
Serum 1,25(OH)_2_D (pg/mL)	15.9 (10.7–22.3)	11.9 (8.1–15.2)	17.8 (12.5–27.0)	<0.001
eGFR (mL/min/1.73 m^2^)	27 (17–38)	20 (16–30)	30 (18–41)	<0.001
Urinary albumin (mg/g Cr)	2134 (962–3731)	2217 (1198–3726)	1983 (929–3723)	0.235
ERI (µg/kg/g/dL/month)	0.14 (0.09–0.21)	0.25 (0.22–0.34)	0.11 (0.08–0.14)	<0.001
hsCRP (mg/dL)	0.7 (0.5–1.0)	0.9 (0.8–1.1)	0.6 (0.4–0.9)	<0.001

Values are expressed as median (interquartile range) or number (%). BMI, body mass index; CERA, continuous erythropoietin receptor activator; DBP, diastolic blood pressure; eGFR, estimated glomerular filtration rate; ERI, erythropoietin resistance index; ESA, erythropoiesis-stimulating agent; hsCRP, high-sensitivity C-reactive protein; iPTH, intact parathyroid hormone; SBP, systolic blood pressure; 25(OH)D, 25-hydroxyvitamin D; 1,25(OH)_2_D, 1,25-dihydroxyvitamin D. ERI-based relative ESA hyporesponsiveness was operationally defined as the highest tertile of ERI within the study cohort.

**Table 2:** Univariable and multivariable analyses of factors associated with relative ESA hyporesponsiveness in the study subjects (*n* = 363).

	Univariable		Multivariable	
	Odds Ratio (95% CI)	*p*	Odds Ratio (95% CI)	*p*
Age (1 year)	0.99 (0.98–1.01)	0.695	1.01 (0.99–1.02)	0.414
Sex (male)	1.12 (0.73–1.74)	0.603	1.47 (0.83–2.59)	0.186
Diabetes mellitus	1.28 (0.83–1.99)	0.266		
BMI (1 kg/m^2^)	1.01 (0.98–1.05)	0.470		
SBP (10 mmHg)	0.98 (0.92–1.06)	0.649		
DBP (10 mmHg)	1.04 (0.93–1.16)	0.491		
Serum albumin (1 g/dL)	1.08 (0.67–1.75)	0.745		
Serum calcium (1 mg/dL)	1.21 (0.74–1.99)	0.443		
Serum phosphate (1 mg/dL)	1.05 (0.84–1.32)	0.679		
Total cholesterol (1 mg/dL)	1.00 (0.99–1.01)	0.189		
Transferrin saturation (1%)	0.92 (0.90–0.95)	<0.001	0.93 (0.90–0.97)	<0.001
Ferritin (10 ng/mL)	0.96 (0.94–0.98)	<0.001	0.97 (0.95–0.99)	0.001
Serum iPTH (10 pg/mL)	1.14 (1.09–1.19)	<0.001	1.08 (1.02–1.13)	0.007
Serum 25(OH)D (1 ng/mL)	1.01 (0.98–1.04)	0.465		
Serum 1,25(OH)_2_D (1 pg/mL)	0.88 (0.85–0.92)	<0.001	0.88 (0.85–0.92)	<0.001
eGFR (1 mL/min/1.73 m^2^)	0.96 (0.94–0.98)	<0.001	0.99 (0.97–1.02)	0.591
Urinary albumin (100 mg/g Cr)	1.01 (0.99–1.02)	0.312		
hsCRP (1 mg/dL)	3.11 (1.91–5.06)	<0.001	2.35 (1.38–4.01)	0.002

BMI, body mass index; DBP, diastolic blood pressure; eGFR, estimated glomerular filtration rate; ESA, erythropoiesis-stimulating agent; hsCRP, high-sensitivity C-reactive protein; iPTH, intact parathyroid hormone; SBP, systolic blood pressure; 25(OH)D, 25-hydroxyvitamin D; 1,25(OH)_2_D, 1,25-dihydroxyvitamin D. ERI-based relative ESA hyporesponsiveness was operationally defined as the highest tertile of ERI within the study cohort. Blank cells indicate variables not included in the final multivariable model.

**Table 3:** Univariable and multivariable analyses of factors associated with ERI in the study subjects (*n* = 363).

	Univariable		Multivariable	
	*ρ*	*p*	*β*	*p*
Age (year)	0.02	0.772	0.06	0.165
Sex (male)	−0.00	0.933	0.03	0.523
Diabetes mellitus	0.07	0.190		
BMI (kg/m^2^)	0.02	0.661		
SBP (mmHg)	−0.01	0.855		
DBP (mmHg)	0.03	0.538		
Serum albumin (g/dL)	−0.01	0.899		
Serum calcium (mg/dL)	0.00	0.967		
Serum phosphate (mg/dL)	0.00	0.935		
Total cholesterol (mg/dL)	0.02	0.662		
Transferrin saturation (%)	−0.25	<0.001	−0.18	<0.001
Ferritin (ng/mL)	−0.18	<0.001	−0.13	0.005
Serum iPTH (pg/mL)	0.34	<0.001	0.18	<0.001
Serum 25(OH)D (ng/mL)	0.04	0.477		
Serum 1,25(OH)_2_D (pg/mL)	−0.45	<0.001	−0.36	<0.001
eGFR (mL/min/1.73 m^2^)	−0.28	<0.001	−0.06	0.231
Urinary albumin (mg/g Cr)	0.02	0.637		
hsCRP (mg/dL)	0.36	<0.001	0.15	0.001

BMI, body mass index; DBP, diastolic blood pressure; eGFR, estimated glomerular filtration rate; ERI, erythropoietin resistance index; hsCRP, high-sensitivity C-reactive protein; iPTH, intact parathyroid hormone; SBP, systolic blood pressure; 25(OH)D, 25-hydroxyvitamin D; 1,25(OH)_2_D, 1,25-dihydroxyvitamin D. *ρ*, Spearman’s rank correlation coefficient; *β*, standardized regression coefficient from multivariable linear regression analysis using natural-log-transformed ERI as the dependent variable. Blank cells indicate variables not included in the final multivariable model.

## Data Availability

Data is contained within the article.

## Abbreviations

The following abbreviations are used in this manuscript:

AUC	area under the curve
BMI	body mass index
CERA	continuous erythropoietin receptor activator
CI	confidence interval
CKD	chronic kidney disease
CKD-EPI	Chronic Kidney Disease Epidemiology Collaboration
CKD-MBD	chronic kidney disease–mineral and bone disorder
CVD	cardiovascular disease
DBP	diastolic blood pressure
eGFR	estimated glomerular filtration rate
ERI	erythropoietin resistance index
ESA	erythropoiesis-stimulating agent
hsCRP	high-sensitivity C-reactive protein
iPTH	intact parathyroid hormone
IQR	interquartile range
KDIGO	Kidney Disease: Improving Global Outcomes
OR	odds ratio
PTH	parathyroid hormone
ROC	receiver operating characteristic
SBP	systolic blood pressure
VDR	vitamin D receptor
25(OH)D	25-hydroxyvitamin D
